# Impact of early initiation versus national standard of care of antiretroviral therapy in Swaziland’s public sector health system: study protocol for a stepped-wedge randomized trial

**DOI:** 10.1186/s13063-017-2128-8

**Published:** 2017-08-18

**Authors:** Fiona J. Walsh, Till Bärnighausen, Wim Delva, Yvette Fleming, Gavin Khumalo, Charlotte L. Lejeune, Sikhathele Mazibuko, Charmaine Khudzie Mlambo, Ria Reis, Donna Spiegelman, Mandisa Zwane, Velephi Okello

**Affiliations:** 1 Clinton Health Access Initiative, Boston, MA USA; 2 Harvard T.H. Chan School of Public Health, Boston, MA USA; 3 Africa Health Research Institute (AHRI), Mtubatuba, South Africa; 4 Institute of Public Health, University of Heidelberg, Heidelberg, Germany; 5 The South African Department of Science and Technology - National Research Foundation (DST-NRF) Centre of Excellence in Epidemiological Modelling and Analysis (SACEMA), Stellenbosch University, Stellenbosch, South Africa; 6 Hasselt University, Center for Statistics, Diepenbeek, Belgium; 7 Ghent University, International Centre for Reproductive Health, Gent, Belgium; 8 KU Leuven, Rega Institute for Medical Research, Leuven, Belgium; 9 aidsfonds, Amsterdam, The Netherlands; 10 Swaziland National Network of People Living with HIV/AIDS (SWANNEPHA), Mbabane, Swaziland; 11 Clinton Health Access Initiative, Mbabane, Swaziland; 12 Ministry of Health, Mbabane, Swaziland; 13 University of Amsterdam, Amsterdam, The Netherlands; 14 Leiden University Medical Center, Leiden, The Netherlands; 15 Children’s Institute, University of Cape Town, Cape Town, South Africa; 16 SAfAIDS, Harare, Zimbabwe

**Keywords:** Antiretroviral treatment, Swaziland, HIV/AIDS, Prevention

## Abstract

**Background:**

There is robust clinical evidence to support offering early access to antiretroviral treatment (ART) to all HIV-positive individuals, irrespective of disease stage, to both improve patient health outcomes and reduce HIV incidence. However, as the global treatment guidelines shift to meet this evidence, it is still largely unknown if early access to ART for all (also referred to as “treatment as prevention” or “universal test and treat”) is a feasible intervention in the resource-limited countries where this approach could have the biggest impact on the course of the HIV epidemics. The *Max*ART Early Access to ART for All (EAAA) implementation study was designed to determine the feasibility, acceptability, clinical outcomes, affordability, and scalability of offering early antiretroviral treatment to all HIV-positive individuals in Swaziland’s public sector health system.

**Methods:**

This is a three-year stepped-wedge randomized design with open enrollment for all adults aged 18 years and older across 14 government-managed health facilities in Swaziland’s Hhohho Region. Primary endpoints are retention and viral suppression. Secondary endpoints include ART initiation, adherence, drug resistance, tuberculosis, HIV disease progression, patient satisfaction, and cost per patient per year.

Sites are grouped to transition two at a time from the control (standard of care) to intervention (EAAA) stage at each four-month step. This design will result in approximately one half of the total observation time to accrue in the intervention arm and the other half in the control arm. Our estimated enrolment number, which is supported by conservative power calculations, is 4501 patients over the course of the 36-month study period.

A multidisciplinary, mixed-methods approach will be adopted to supplement the randomized controlled trial and meet the study aims. Additional study components include implementation science, social science, economic evaluation, and predictive HIV incidence modeling.

**Discussion:**

A stepped-wedge randomized design is a causally strong and robust approach to determine if providing antiretroviral treatment for all HIV-positive individuals is a feasible intervention in a resource-limited, public sector health system. We expect our study results to contribute to health policy decisions related to the HIV response in Swaziland and other countries in sub-Saharan Africa.

**Trial registration:**

ClinicalTrials.gov, NCT02909218. Registered on 10 July 2016.

**Electronic supplementary material:**

The online version of this article (doi:10.1186/s13063-017-2128-8) contains supplementary material, which is available to authorized users.

## Background

The global community has made extraordinary strides in scaling up treatment for people living with HIV/AIDS over the past two decades. Since 2000, there has been a 30-fold increase in people accessing antiretroviral therapy (ART) globally from 250,000 people to more than 17 million people in 2015 [1, 2]. The rate of new infections globally has also come down in the last decade from 3.3 million new infections in 1998 to 2.1 million in 2015 [2]. This success has been driven, in part, by an unprecedented increase in financial resources, with annual funding levels increasing from US$5 billion in 2003 to US$19.2 billion in 2014 [3].

However, despite the significant progress, the global community is still far from ensuring that all people living with HIV/AIDS (PLHIV) eligible for treatment are receiving it. Moreover, without a significant reduction in new infections, the number of individuals in need of treatment will continue to expand, as will the costs of HIV treatment programs [4, 5]. Identifying and implementing effective prevention interventions to try and get ahead of the epidemic is a crucial next step in the global response to HIV.

Over the past several years, HIV research has demonstrated that earlier and expanded access to ART could have a significant impact on HIV incidence. Results from the HIV Prevention Trials Network 052 (HPTN 052) trial showed that early access to ART prevents onward transmission of the virus to the uninfected partner in heterosexual HIV-discordant couples. In 2011, the trial reported not only a 96% decrease in HIV transmission, but also a 41% decrease in HIV-related morbidity from early initiation of ART [6]. The results from the Strategic Timing of Antiretroviral Therapy (START) trial in 2015 demonstrated that the immediate initiation of ART was beneficial for morbidity and mortality with no increased rate of adverse effects [7]. In response to this growing body of epidemiological evidence, World Health Organization (WHO) 2015 treatment guidelines recommended ART initiation for all HIV-positive individuals [8].

While the clinical impact of Early Access to ART for All (EAAA) is evident, critical implementation questions remain. Recently, results from the Treatment as Prevention (TasP) Trial in rural KwaZulu-Natal in South Africa did not demonstrate a difference in HIV incidence between communities randomized to either immediate offer of ART for all HIV-positive individuals compared to the standard of care [9]. The study results demonstrated not only the need for high rates of linkages to care following diagnosis, but also the importance of understanding how to best deliver early ART in southern Africa. As the latest WHO guidelines are recommending EAAA [10] and countries in sub-Saharan Africa start to follow this recommendation [11], the critical question is no longer whether EAAA should be implemented, but rather how to implement it in resource-limited settings where the epidemic is most prevalent.

It remains unknown, however, what the impact of EAAA policies will be on public sector health systems in sub-Saharan Africa. The overall HIV treatment program effectiveness in successfully treating HIV patients could change, as could patient satisfaction, patients’ economic welfare, and provider satisfaction. Policymakers in high HIV prevalence countries and stakeholders in the international community require empirical evidence to assess the feasibility and acceptability of EAAA policies, as well as the clinical outcomes, affordability, and scalability of national implementation of this intervention through routine, public sector health systems.

This study was designed in response to questions posed by Swaziland’s policymakers about the impact of an EAAA policy on their national HIV/AIDS care and treatment program. The study aims to generate the evidence needed to more fully understand what is required to successfully implement an EAAA strategy in a public sector health system in sub-Saharan Africa.

## Specific aims

This trial aims to:

1. quantify the causal impact of early access to ART for all HIV-infected adults, irrespective of CD4 count or clinical staging, on ART retention and viral suppression in a public sector health system;
2. quantify the causal impacts of EAAA on average health care expenditures and resources use;
3. quantify the causal impacts of EAAA on patient satisfaction, patients’ welfare, and provider satisfaction;
4. to establish the feasibility and acceptability of EAAA;
5. establish the role of PLHIV, traditional leaders, civil society, and communities for implementing EAAA;
6. determine the social and institutional processes throughout EAAA implementation to help interpret differences in health service delivery and patient experiences of ART initiation, adherence, and retention before and after the intervention;
7. estimate the potential change in HIV incidence and ART program size if EAAA were to be implemented nationwide in Swaziland; and
8. predict the long-term cost-effectiveness of EAAA.

## Methods

### Study design

**Fig. 1:**
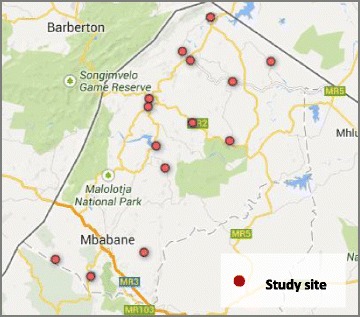
Map of study sites in Hhohho Region

This is a randomized seven-stepped-wedge design on seven pairs (13 government clinics and one regional hospital) in Hhohho Region (see Fig. 1) with open enrollment for all adults aged ≥ 18 years.

The study sites include a mix of high-volume and low-volume facilities (volume of patients) that are already providing a comprehensive package of HIV care and treatment services per Swaziland’s national adult HIV treatment guidelines [12].

**Fig. 2:**
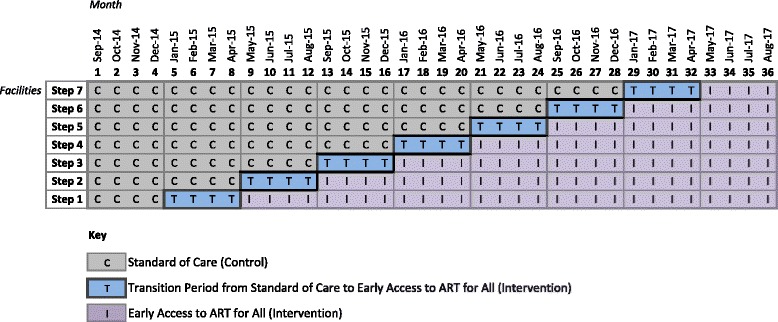
Stepped-wedge study design

Sites are grouped to transition two at a time from the control (national eligibility guidelines or current standard of care) to the intervention (EAAA) stage every four months. As illustrated in Fig. 2, the study will enroll all eligible HIV-positive patients who arrive at the facility in Month 1. All sites will start in the standard of care stage (“C”), and then each site will have a four-month transition period (“T”) to transition consecutively until all sites are in the intervention (“I”) stage. The sites will start implementing the intervention on the first day of the transition period.

Individuals who arrive at one of the study facilities during the control (“C”) stage will be offered ART according to the current national guidelines. Sites will follow the current national standard of care for HIV care and treatment while in the control stage.

**Fig. 3:**
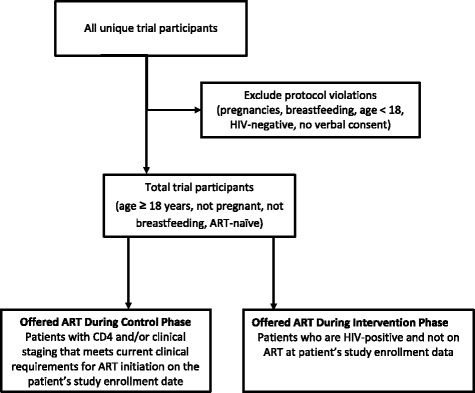
Trial flow diagram

During the transition period (“T”) and the intervention (“I”) stage, all HIV-positive patients will be offered ART per the EAAA intervention (an offer of immediate ART initiation irrespective of CD4). Study participants who were already in pre-ART at the start of the study or who were enrolled into pre-ART during the control stage according to national standard of care will be offered ART at their first follow-up appointment at their enrollment site following the site’s transition to the intervention stage. This way, everyone who is enrolled into the study population will eventually be offered the EAAA intervention (Fig. 3).

An additional file provides the protocol checklist (see Additional file 1. SPIRIT checklist for the *Max*ART EAAA Trial, indicating which manuscript page contains each element of the study protocol).

### Setting

Swaziland faces a prevalence rate of 31–32% among its 18–49-year-olds, but the epidemic disproportionately affects women [13]. The recent Swaziland HIV Incidence Measurement Survey highlighted that overall HIV incidence is higher among women (3.1% for women and 1.7% for men) and highest of all in women 20–24 years of age (4.2%) [13].

The Swaziland National AIDS Program (SNAP) has been in existence since 2004 and has scaled up its program each year, resulting in a significant increase in the number of people on treatment. The country has made great strides in their HIV response, including a robust national ART program and expanding the immunological treatment eligibility criteria in their national HIV treatment guidelines since 2010 to individuals with CD4 < 350 cells/mm^3^. In December 2015, the country further expanded its eligibility criteria to CD4 < 500 cells/mm^3^ as per the WHO 2015 guidelines. At the end of December 2015, 147,274 adults were on treatment, which represents more than 90% treatment coverage based on current eligibility criteria (CD4 count > 500 cell/mm^3^) and estimates of need [14].

### Trial participants (inclusion and exclusion criteria)

All HIV-positive individuals who are aged 18 years or older and are ART naïve—excluding pregnant or breastfeeding women—who attend the health facilities included in the study will be asked for their verbal consent to enroll in the study. Those individuals whose CD4 is greater than the current national guidelines for ART initiation and whose WHO stage is not 3 or 4 are the primary study population. Written informed consent will be secured for participation in social science research and the economic evaluation.

Participants must be able and willing to give verbal consent for trial participation. Individuals considered unable to provide and participate in informed consent include those with uncontrolled psychiatric disorders or severe neurological impairment.

### Sample size

The primary study population will be 2008 newly diagnosed or returning pre-ART patients as defined by the current national guidelines who are enrolled during the control or the intervention stage over the 36-month study period. In addition, 2493 patients with CD4 ≤ 350 cells/mm^3^ will be enrolled to form a total study population of 4501. With recent changes in Swaziland’s national guidelines, the number of patients enrolled within the primary population is expected to be lower than projected due to the change in ART initiation threshold.

The trial statisticians performed randomization of the study sites before the start of the study. The 14 sites have been grouped into seven pairs: four pairs were grouped according to geographic proximity; two pairs were grouped to coordinate the timing with operational or logistical issues; and one pair was grouped and excluded from the randomization so that it can transition to the intervention first to accommodate the social science research timeline. The six remaining pairs will be randomized into steps 2 to 7 when each site transitions to the intervention stage, but the health workers, study participants, and study teams are blinded to the timing to minimize bias.

### Power calculations

Power calculations were undertaken to ensure that the minimal detectable difference in the two primary endpoint rates, given that the projected sample size was well within the expected given prior literature [15]. For one-year retention, 1160 patients in the primary study population were projected to enroll during the EAAA (intervention) phase and 2088 under the control phase in the first 24 months to achieve a one-year follow-up.

Using the standard method for power calculations for binary endpoints in stepped-wedge designs, with a 5% two-tailed Type I error rate, an 80% power or more was found to detect a 6–8 percentage point increase in the one-year retention rate over a range of scenarios considered for the one-year retention rate in SOC from 40–80% and an intra-cluster correlation coefficient (ICC) of 0–0.5, with no prior data available in Swaziland to allow ICC estimation. It is well-known that the stepped-wedge design is insensitive to the assumed value of the ICC [15]. To be conservative, we considered a wide range. Also, here, the minimum detectable effect sizes did not vary over the range of ICCs considered.

For the six-month viral load suppression after ART initiation endpoint, 1869 patients were projected to enroll in the EAAA (intervention) phase and 1728 in the control phase over the first 30 months to ascertain a six-month viral load after ART initiation. Following the methods as above, we found that there was 80% power or more to detect a 7–8 percentage point increase in the six-month viral suppression rate over a range of scenarios considered for the six-month viral suppression rate in standard of care of 20–70% and an intra-cluster correlation coefficient of 0.10–0.20.

#### Study procedures

##### Community sensitization

Community mobilization is used to sensitize communities on early ART and create demand for uptake of services. A comprehensive messaging strategy was designed to address the complex personal, cultural, social, and sexual considerations that influence decisions around HIV testing and ART initiation. Messages are delivered through a variety of methods and activities, including sensitization meeting with key community actors, including traditional leaders to gain their support. Community dialogues targeting community members are conducted using edutainment providing HIV testing services for active case finding at the community level.

Door-to-door visits where information, education, and communication materials are delivered by trained community-based volunteers. Community-based HTC is provided during the community events and linkage to care is ensured through working with expert patients and HIV testing partners. Support groups are equipped with skills and tools to provide treatment literacy to PLHIV. These activities are conducted within a 20–30-km radius around each study site.

##### Clinical procedures for ART initiation and follow-up

The study aims to promote a continuum of care and to evaluate feasibility of an EAAA strategy within a public sector health system setting. Therefore, the study will be implemented in a public sector health system where there is already an essential package of services available through the current national ART program. The main differences in the package of services delivered in the standard of care vs. the intervention stage of the study are outlined in Table 1 below.

**Table 1:** Key differences in package of services in control vs. intervention stage for patients enrolled in the EAAA study

Service	Control	Intervention
Eligibility criteria for ART initiation	Current national treatment guidelines	All HIV-positive individuals who are aged 18 years or older, excluding pregnant or breastfeeding women, who attend the health facilities will be offered ART regardless of CD4.
Pre-ART counseling sessions	2–3 sessions prior to ART initiation	Same-day counseling and ART initiation if patient is ready. Continue counseling after initiation.

Additional blood investigations will be conducted to inform the measurement of the study endpoint, viral load suppression, but these lab tests will be taken in both the standard of care and intervention groups. Aside from the eligibility criteria and the messaging used during pre-ART counseling sessions, there will be no differences in the package of follow-up services provided to patients between the control and intervention study groups.

Per national guidelines, ART initiation takes place within four weeks of HIV testing or two weeks of enrollment at the trial clinic, unless delayed per clinician’s guidance. All ART drugs used in the trial follow the Swaziland adult HIV management guidelines. All individuals who are eligible for treatment will be initiated on Swaziland’s recommended first-line ART regimen (TDF + 3TC + EFV), unless contraindicated when recommended alternate regimens will be used per national guidelines. These alternatives are: TDF + 3TC + NVP or AZT + 3TC + NVP (when EFV cannot be used); ABC + 3TC + EFV or AZT + 3TC + EFV (when TDF cannot be used); ABC + 3TC + EFV or d4T + 3TC + EFV (when AZT cannot be used).

All patients in the control and intervention phase will follow the nationally recommended follow-up schedule for individuals who are on ART or enrolled in pre-ART.

##### Laboratory procedures

Routine viral load and drug resistance testing are important to understand the implications and impact of the interventions. Patients in both study groups will receive routine viral load and drug resistance testing in addition to the routine standard of care laboratory tests per the recommendation of and as dictated by Swaziland’s National Comprehensive HIV Package of Care, including: HIV test; CD4 test, full blood count; AST/ALT; creatinine; and Hepatitis B surface antigen.

Each patient will provide samples every six months for viral load testing. For drug resistance testing, a pre-treatment sample will be collected from each patient upon study enrollment and at ART initiation. Upon initial signs of ART failure (two consecutive viral load >1000 copies/mL), a post-treatment sample will be collected for drug resistance testing.

##### Data collection procedures

The data for the clinical endpoints will be collected at the facility on an ongoing basis as individuals are enrolled into the study and return for follow-up visits at the facility. Health workers will collect data from individual study participants at each site using the national chronic care file paper-based data collection forms.

Where possible, the study will collect data from existing data sources (i.e. patient’s chronic care files, facility registers) to inform the research. Data will also be collected from individual study participants at each visit. For data collected on-site outside of existing Swaziland Ministry of Health procedures, paper-based data collection forms will be used. Two copies of this form are kept: one copy in the patients’ clinical file and the second copy as part of the study records.

An electronic database will also be developed to organize all study-related data. Data clerks will be responsible for the daily entry of data from paper forms into the electronic database. All data will be encrypted to ensure patient confidentially before, during, and after the trial. The principal investigator, study statistician, and data manager will have access to the final trial dataset.

##### Care of patients at the end of the trial

Individuals enrolled in this study and initiated on ART will continue to receive care and treatment through Swaziland’s public sector health system for the rest of their lives.

## Trial outcomes

A multidisciplinary, mixed-methods approach will be adopted to meet the study aim, including implementation science research, social science research, economic evaluations and cost-effectiveness modeling, and incidence modeling.

### Implementation science

This is one of the first studies in the treatment-as-prevention field that has been designed to answer critical implementation questions (i.e. acceptance and retention among patients initiated on ART at higher CD4 thresholds), and determine the “real world” potential of this new prevention intervention [9, 16–19]. In resource-limited countries, ministries of health need guidance on how to aggressively and effectively adopt the new guidelines and targets, and to understand what the changes will mean for their programs and budgets. As such, this study’s primary and secondary outcomes (Table 2) are structured to assess the feasibility, acceptability, clinical outcomes, affordability, and scalability of offering early antiretroviral treatment to all HIV-positive individuals in Swaziland’s public sector health system.

**Table 2:** Primary and secondary outcomes

Primary outcomes	Definition
Retention	Alive and in care at each 12-month time point following enrollment
Viral suppression	The proportion of individuals alive and in care whose viral load is below 1000 copies/mL (virally suppressed) after six months on treatment
Secondary outcomes	Definition
Retention	At each six-month time point following enrollment
Viral suppression	At each six-month time point following enrollment
Mortality	At each six-month time point following enrollment
Visit adherence among those initiated on ART	Proportion of missed visits as a number of scheduled appointments among ART-ineligible patients by end of follow-up
Drug resistance	Proportion of drug resistance among ART-ineligible patients with two virological failures who have received genotype resistance testing
Tuberculosis	Proportion of ART-ineligible patients diagnosed with tuberculosis following enrolment (recurrent and new incident)
ART uptake among those who are eligible	Proportion of HIV-positive individuals who are eligible for initiation who are successfully initiated to ART within on the day of, within two weeks, and one and three months of becoming eligible
Cost per patient per year	Average public and private healthcare expenditures per patient
Patient satisfaction	Average patient satisfaction
Provider satisfaction	Average job satisfaction among the professionals providing HIV treatment
Patients’ employment, income, and education	Average patients’ employment, income, and educational attainment

### Social science research

Social science research methods are used to triangulate the study’s findings on ART initiation, adherence, and retention; to help identify effect moderators by analyzing throughout the implementation social and structural factors and differences in health service delivery that may differently affect the uptake of services before and after facilities have transitioned to EAAA; and to contribute to a deeper understanding of reasons for delayed initiation, non-adherence, and non-retention. These objectives will be met by conducting mixed-methods research in a sample of nine study sites including a random quantitative survey, semi-structured interviews with health providers and patients, and participant observation.

The quantitative survey will be conducted with a random sample of patients tested HIV-positive during the control phase and during the intervention phase at six and 12 months (T1, T2, T3, n = 380 per survey, 760 in total). The baseline survey will be conducted in 12 months and will include a random sample of HIV-positive patients who have initiated ART less than 12 months prior to the interview. A follow-up survey will be conducted six months after the site’s last day of its first transition month with patients who were initiated within six months of the site’s transition to the EAAA strategy (intervention stage). The second follow-up survey at 12 months after the site last day of its first transition months will allow for the comparison of the experiences of those being initiated on ART and adherence before and after the intervention, at six months and at 12 months of being on ART. Changes across time of distal/proximate factors will be studied, including sex, age, family context, socioeconomic status, employment status, health-seeking history, gendered decision-making patterns, and illness experience. Outcomes will include ART initiation, adherence and retention, patients’ experience of immediate ART initiation process (i.e. confidentiality, informed consent, enacted stigma, disclosure strategies, and sexual and reproductive health desires).

To assess health providers’ experiences and attitudes about EAAA, semi-structured interviews will be conducted with a limited number at two points in time: the second month into the transition and then again four months after the last transition month (information-rich case sampling of those most involved in EAAA). To understand reasons for delayed initiation, non-adherence, and non-retention, qualitative in-depth interviews will be conducted among a diverse sample of 50 HIV-positive individuals (ten interviews per category, of which five are men and five are women) who have initiated ART. These interviews will take place at random points of time during the intervention stage at different stages of each individual’s cascade of care and treatment. The categories include EAAA patients who delayed or refused ART initiation and, identified through the EAAA database, patients who are not virally suppressed after six months on treatment, patients who are virally suppressed after six months on treatment, patients who have not been retained in care/on ART at six months, and patients who have been retained in care/on ART at six months.

Finally, at each of the sampled facilities, three weeks of observation will be conducted of pre- and post-test counselling, ART initiation, and adherence counselling: at baseline before the site transitions to the intervention stage, in the second month during transition, and seven months after the first transition month. These observations will take place simultaneously with the baseline patient semi-structured survey interview, health provider, and/or qualitative patient interviews to limit the time spent at each site.

### Economic evaluation

The *Max*ART study aims to quantify empirically the causal impacts of EAAA on a range of important economic outcomes, including healthcare expenditure, resources use, and patients’ welfare. In addition, the cost-effectiveness of the EAAA approach will be estimated through a predictive economic evaluation. To this end, a comprehensive costing study will be conducted, which includes systematic assessments of both the government and private activities for EAAA and the unit costs for these activities. As part of the costing study, the national health sector budgets and expenditure reports will be regularly reviewed and the data relevant to EAAA will be recorded, such as provider salaries and facility, supply chain, drug, and laboratory costs. A time-and-motion study will also be conducted to determine how much time healthcare providers spend on the different HIV treatment-related activities, including consultation time, physical examination, counseling, and interpreting laboratory results. Finally, patient exit interviews will be used to measure private healthcare expenditure and the patient and provider time required under a EAAA protocol.

### Incidence modeling

To inform the national policymaker’s interpretation of the stepped-wedge trial, as well as long-term planning of a national EAAA program, a mathematical model has been developed to simulate the heterosexual transmission of HIV and the provision of ART in the catchment area of the study sites and for Swaziland as a whole [20].

The objectives of the HIV incidence modeling component of this study are twofold. First, a narrow, data-driven simulation study will be conducted to estimate the impact of the EAAA stepped-wedge trial on HIV incidence during the study period in the catchment area of the study sites. Second, a larger, projection study will be conducted to estimate the impact of a national EAAA program on HIV incidence over three time horizons: (1) five years, (2) ten years, and (3) 15 years (Oct 2016–Sept 2031) for the entire country of Swaziland.

Outcome indicators for both analyses are the incidence rate ratio, number of HIV infections averted, the number of HIV-negative life-years gained, the number of life-years gained, and the number of additional person-years of ART provided (only for the national-level analysis). Outcome indicators for the first analysis will be calculated using model output from both the control and EAAA phases of the clinics, weighted for the population size of the clinic catchment area and the amount of time spent in each phase. Besides population-average outcome indicators, the outcome indicators stratified by age group and gender will also be calculated.

For the estimation of the impact of a national EAAA program on HIV incidence, two scenarios will be contrasted against one another. In the control scenario, the country will maintain its current national treatment guidelines for the entire simulation period. In the EAAA scenario, a shift to universal treatment will be simulated from October 2016 onwards. The rates of HIV diagnosis, ART initiation, viral suppression, and program retention among HIV-positive patients with CD4 cell counts > 500/mL in the intervention scenario will be equal to those inferred from the narrow, data-driven simulation study.

The epidemiological model will be calibrated to historic data on key demographics (population growth rate, crude birth and death rates, geographical population density), historic HIV prevalence data (stratified by age group and gender), historic ART program size data (number of people on ART, by age group and gender), and recent HIV incidence data, by age group and gender [13]. As parameters for the sexual behavior in the model population are typically difficult to estimate directly from empirical data, due to selection bias and social desirability bias in sexual behavior surveys, these model parameters will be inferred through an iterative Approximate Bayesian Computation algorithm with wide prior distributions [21].

Once the “model world” is deemed sufficiently resembling of the real world, the “model trial” will be calibrated to the real trial by adjusting the hazard functions for initiating ART and dropping out of the ART program in order to match key summary statistics: the size of the ART patient population by clinic, age group, gender, baseline viral load, and baseline CD4 cell count. In addition, the percentage of patients that is virally suppressed six or more months after ART initiation will be matched across all sites, during the period that the sites were offering EAAA, as well as the percentage of patients that are virally suppressed across all sites, during the period that the sites were in the control phase.

## Analysis

The primary analysis will follow the intent to treat approach, with each patient’s intervention status assigned as the one in place at their facility at the time of study enrollment. The log rank test, stratified by step time, will be used to assess the statistical significance of any differences observed in one-year retention and six-month suppression rates between the standard of care and early access groups. Kaplan–Meier curves will be produced and used to obtain point and interval estimates of the cumulative incidence of the two primary endpoints at the landmark time points. In the retention analysis, censoring will occur only by administrative end of follow-up or one year from enrollment, whichever happens first. In the viral suppression analysis, only patients initiated to ART who have at least six months of follow-up at the time of administrative end of follow-up and at least one viral load measured after that time will be included. Thus, this endpoint will be interpreted as viral suppression given comprehensive clinical retention.

Censoring for this endpoint occurs at the minimum of the date of the last viral load six months after ART initiation, the administrative end of follow-up, and date of death, whichever happens first. Secondary analysis of the primary endpoints will include estimation of relative risks, their 95% confidence intervals, and *p* values for the intervention effects using the Cox proportional hazards model, adjusting for potential measured confounders, including facility, sex, age, BMI, disclosure to partner, occupation, education, baseline CD4, baseline viral load, date of first HIV-positive test, baseline opportunistic infections, baseline WHO stage, and baseline tuberculosis history. Additional analysis will adjust for those variables which are repeatedly updated over time as time-varying covariates, and will, in addition, consider patient’s intervention status as time-varying, allowing for the transition of facilities over time, as well as a time-varying eligibility status, allowing for changes in national guidelines and patient preferences.

Marginal structural models will be explored to adjust for possible bias due to dependent censoring [22–24]. Modification of the intervention effects by age, sex, baseline disease stage, facility type, and other hypothesized modifiers will be explored by testing for interaction using the partial likelihood ratio test and providing point and interval estimates of stratum-specific intervention effects. Longitudinal analysis of continuous variables such as BMI, CD4, and viral load in relation to differences in the trajectories of change in these variables over time by intervention group will be conducted under the generalized estimating equations framework, following similar modeling strategies as described above [24].

Since one of our primary outcomes is retention, where attrition can be considered the opposite of retention, the intent to treat analysis is ideally situated to assess the impact of the intervention on retention/attrition and cannot be biased by it. However, six-month viral suppression can be biased by differential non-retention. Therefore, in secondary analysis, as recommended by Little et al. [25], we will conduct a sub-study of non-retained clients to better understand the reasons for this and to improve their classification as dead, transferred, or truly lost to follow-up for planned secondary analyses.

## Discussion

The *Max*ART trial is designed to better understand the “real world” complexities of implementing this treatment for all approach. The quantitative and qualitative social science research produced from this study will be essential to understand the social and behavioral impact of offering ART to HIV-positive individuals soon after diagnosis. Community perception of the intervention and research on how to best engage communities to support this intervention will have an important influence on uptake, linkages to care, retention, and adherence. Further, a comprehensive economic evaluation to assess how expanding access to treatment will improve livelihoods and productivity and, ultimately, decrease the cost to the health system as a whole. Modeling will be used to also understand the cost-effectiveness of the intervention, which is key evidence for the Government of Swaziland’s national financial planning. This study has been designed to answer the critical implementation questions posed by the Government of Swaziland as the country works towards rolling out a treatment for all approach across the country to turn the tide on their epidemic.

## Trial status

This trial is underway. Clients will be enrolled into the study through August 2017.

## Additional file

Additional file 1: SPIRIT Checklist *Max*ART Trial. SPIRIT checklist for the *Max*ART EAAA Trial, indicating which manuscript page contains each element of the study protocol. (DOCX 53 kb)

## Abbreviations

3TC: Lamivudine
AIDS: Acquired immunodeficiency syndrome
ART: Antiretroviral treatment
CAB: Community Advisory Board
CD4: Cluster of differentiation 4
DSMB: Data Safety and Monitoring Board
EAAA: Early Access to ART for All
EFV: Efavirenz
FTC: Emtricitabine
HIV: Human immunodeficiency virus
LPV/r: Lopinavir/ritonavir
MOH: Ministry of Health
SNAP: Swaziland National AIDS Program
TB: Tuberculosis
TDF: Tenofovir
VL: Viral load
WHO: World Health Organization
